# Advances in Research on Adolescent Suicide and a High Priority Agenda for Future Research

**DOI:** 10.1111/jora.12614

**Published:** 2021-12

**Authors:** Matthew G. Clayton, Olivia H. Pollak, Sarah A. Owens, Adam Bryant Miller, Mitchell J. Prinstein

**Affiliations:** University of North Carolina at Chapel Hill

**Keywords:** adolescence, resilience, risk factors, Suicide

## Abstract

Suicide is the second leading cause of death for adolescents in the United States, yet remarkably little is known regarding risk factors for suicidal thoughts and behaviors (STBs), relatively few federal grants and scientific publications focus on STBs, and few evidence-based approaches to prevent or treat STBs are available. This “decade in review” article discusses five domains of recent empirical findings that span biological, environmental, and contextual systems and can guide future research in this high priority area: (1) the role of the central nervous system; (2) physiological risk factors, including the peripheral nervous system; (3) proximal acute stress responses; (4) novel behavioral and psychological risk factors; and (5) broader societal factors impacting diverse populations and several additional nascent areas worthy of further investigation.

Suicide is a leading cause of death in the United States (Stone et al., 2018). Arguably, no other mental health problem poses a greater risk to human mortality, yet our understanding of pathways through which suicidal thoughts and behaviors (i.e., STBs) develop is relatively limited. Despite significant advances in the past few decades, a recent meta-analysis spanning over 50 years revealed that research examining risk factors for STBs has been remarkably redundant. The same risk factors have been studied repeatedly over the past half-century, and effect sizes for a majority of predictors are fairly low (Franklin et al., 2017). Further, data reveal that in contrast to progress in reducing the burden of several major health disorders (e.g., tuberculosis, pneumonia, and cancer), rates of death by suicide have not declined in the twentieth century (CDC, 2019). In fact, when current rates of death by each of several leading causes are compared to mortality rates over 50 years ago, suicide is among the only cause with increasing, rather than decreasing rates (see Figure 1; data adapted from CDC, 2019).

Notably, suicide is the second leading cause of death among youth ages 10–34 years (CDC, 2019), and epidemiological data suggest that suicide should be considered within a developmental context. Among youth ages 15–24, the rate of completed suicide is over 10 times greater than among youth ages 5–14 (i.e., an increase from 1.3 to 14.3 per 100,000 between these age groups; Kochanek, Murphy, Xu, & Arias, 2019), suggesting that the transition to adolescence represents a unique period of vulnerability for onset of STBs (Miller & Prinstein, 2019). Indeed, approximately one in five adolescents in the United States seriously considers suicide, and seven percent of adolescents attempt suicide, each year (CDC, 2019).

Among adolescents, rates of STBs also differ dramatically by gender, race, and ethnicity. While adolescent males are more likely to die by suicide, adolescent females are almost twice as likely to consider or attempt suicide (CDC, 2017). Data also suggest that adolescents who identify as Native American, White, or Latinx are significantly more likely to report STBs than African American, Asian, Native Hawaiian, or Pacific Islander youth, although this discrepancy in decreasing, as discussed below (e.g., Price & Khubchandani, 2019). In light of these troubling statistics, the Surgeon General, Congress, the Department of Health and Human Services, and the National Institute of Mental Health have called for prioritization of research aimed at advancing understanding of STBs, perhaps especially among high-risk populations (Exec. Order No.13625, 2012; U.S. Department of Health and Human Services, 2010, 2012).

It is therefore unfortunate that compared to work examining STBs among adults, few studies have examined STBs in adolescents. Indeed, until recently (see Bridge, Goldstein, & Brent, 2006; Miller & Prinstein, 2019; Owens, Eisenlohr-Moul, & Prinstein, 2020), few suicide theories have centered around developmental processes that may help explain why STBs increase in the transition to adolescence. In addition, relatively few studies of adolescent STBs have used methodological approaches common in developmental psychopathology research; for example, longitudinal research on youth STBs is rare. Further, within the large body of research on adolescent psychopathology, studies have often lacked specificity, considering STBs as part of related psychopathology (e.g., depression) rather than distinct outcomes in their own right (see Figure 2). Prior work often examines STBs as a unitary construct, despite evidence suggesting unique correlates, risk factors, and treatment approaches for distinct STB outcomes, such as suicidal ideation, plans, and attempts (Nock, 2010). Research on STBs has also infrequently incorporated a focus on neuroscience or biological mechanisms. Lastly, little research has elucidated proximal risk factors for STBs (Franklin et al., 2017), even though identifying factors associated with imminent STBs in youth is a pressing public health priority.

In this “decade in review” article, we discuss several important trends in adolescent STB research that begin to address gaps in this high priority area. Importantly, significant advances in understanding adolescent STBs will require a “neurons to neighborhoods approach” that examines multiple systems and developmental domains (Carey, 2011). This review is accordingly structured to highlight advances in the past 10 years across multiple domains. First, research on recent developmental cognitive neuroscience findings is discussed, highlighting an important potential role of the central nervous system. Next, recent advances examining physiological risk factors (e.g., hormonal variations, menstrual cycle fluctuations) and the peripheral nervous system are reviewed. Proximal risk factors (e.g., physiological responses to acute stressors), a behavioral/psychological risk factor that has received increased empirical attention in relation to STBs (i.e., nonsuicidal self-injury), and broader societal factors impacting diverse populations are discussed next. This review also includes a brief discussion of several additional emerging areas that have been examined in the past decade and warrant further attention (i.e., genetics, real-time monitoring methodologies, complex risk models, social media, sleep, resilience factors, and empirically supported treatments). Each section includes a discussion of relevant theoretical models, notable empirical advances, and suggestions for future directions.

In this review, discrete STB outcomes are discussed based on well-established definitions (Silverman, Berman, Sanddal, O’Carroll, & Joiner, 2007): *suicidal ideation* is the consideration of, or desire to end one’s own life, *suicide attempt* is a deliberate action to end one’s own life, and *suicide death* is a fatal suicide attempt. When studies do not specify STB outcomes, or where multiple outcomes are implicated, we refer more broadly to “STBs”. In this review, STBs does not refer to self-injurious actions performed without suicidal intent (e.g., nonsuicidal self-injury, suicide gestures). While nonsuicidal self-injury (NSSI) is discussed, it is referred to as such and considered distinct from STBs. Last, this review does not include key advances from the past decade that already have been reviewed in recent publications, such as associations among substance abuse, cognitive functioning (e.g., cognitive control, perception), and implicit attitudes toward death or suicide (see Esposito-Smythers & Spirito, 2004; Glenn, Cha, et al., 2017; Glenn, Lanzillo, et al., 2017).

## DEVELOPMENTAL COGNITIVE NEUROSCIENCE AND ADOLESCENT STBs

It is unlikely that complex and multidetermined behaviors, such as STBs, will be predicted strongly by a single risk factor, or result from a deficit in a single developmental competency. More likely is that STBs are associated with transactions across multiple developmental systems. In the past decade, research has begun to suggest that examining the development of, or differences in, underlying neurobiological processes among youth may advance an understanding of acute suicide risk during adolescence.

Compared to childhood and adulthood, adolescence represents a distinct neurodevelopmental stage wherein the adolescent brain undergoes substantial neuroplasticity (Casey, 2015; Galván, 2014). This protracted period of development, which begins around the onset of puberty, lasts into early adulthood and is characterized by sensation seeking (Steinberg, 2008), increased sensitivity to social evaluation and feedback (Somerville, 2013), and increased reactivity to stressors (Dahl & Gunnar, 2009). Maturation in key brain areas during adolescence has helped researchers begin to understand why these behaviors emerge or increase in adolescence. Central to many theoretical models is the idea that subcortical brain regions, including the striatum and amygdala, develop more rapidly than prefrontal cortex (PFC) control regions implicated in self-regulation (for a review, see Casey, 2015). Indeed, research demonstrates that adolescents, relative to children and adults, show greater ventral striatum responses to rewards (Galvan et al., 2006). Further, while adolescents perform as well as adults on certain cognitive tasks, including those that recruit cognitive control processes, adolescents are more easily distracted by appetitive cues (Geier & Luna, 2012; Somerville, Hare, & Casey, 2011). Notably, adolescents’ behavior may be particularly affected in the context of peers (Chein, Albert, O’Brien, Uckert, & Steinberg, 2011; Guyer, Choate, Pine, & Nelson, 2012).

This constellation of increased sensitivity to rewards and social cues, increased emotional reactivity, and decreased self-regulation skills may help to explain onset or increases in psychopathology, including STBs, during adolescence. Early evidence suggests that neural processes implicated in emotion processing, cognitive control, and negative emotional states may be associated with adolescent STBs (for reviews, see Auerbach, Pagliaccio, Allison, Alqueza, & Alonso, 2021 and Schmaal et al., 2020). In a recent review of neural mechanisms and STBs across adult and adolescent samples, Schmaal et al., (2020) suggest that altered structure and functioning in brain regions implicated in excessive negative and blunted positive internal states, negative self-referential thinking, and rumination (e.g., amygdala, hippocampus, precuneus, striatum) may be associated with suicidal ideation. In turn, prefrontal regions associated with cognitive control of emotion and decision-making processes (e.g., dorsomedial and dorsolateral PFC) may play a role in exacerbating these negative internal states, potentially heightening STB risk (Schmaal et al., 2020).

While not specific to adolescents, Schmaal et al., (2020) proposed model can nevertheless be considered alongside other work in developmental psychopathology and studies of adolescent STBs, including those examining adolescents’ responses to acute stressors that have been shown to predict STBs. Research demonstrates that adolescents show greater physiological reactivity to stressors compared to children and adults (Stroud et al., 2009). Peripheral stress responses, such as hypothalamic–pituitary–adrenal (HPA) axis reactivity, activate following exposure to a stressor. The intensity and duration of these responses are governed within subcortical structures of the brain such as the amygdala, which monitors the environment for salient cues signaling reward or threat. Reactivity in these subcortical structures is thought to be modulated via PFC regions, including the ventrolateral and dorsolateral PFC via higher order cognitive strategies (e.g., cognitive reappraisal; Buhle et al., 2014; Ochsner et al., 2004). Of note, these regions overlap with the brain circuitry model of STBs proposed by Schmaal et al., (2020).

In the past decade, preliminary functional neuroimaging (fMRI) research has begun to explore activity in these neural networks among suicidal adolescents. A recent fMRI study of adolescents (*n* = 49) demonstrated that youth with (vs. without) suicidal ideation histories showed reduced activation in the dorsolateral PFC when passively viewing negative stimuli (Miller et al., 2018). Another study including adolescents and young adults with bipolar disorder (*n* = 46) revealed decreased gray matter volumes in the hippocampus, orbitofrontal cortex, and cerebellum among those with (vs. without) suicide attempt histories (Johnston et al., 2017). Additionally, as compared to those without prior attempts, youth with suicide attempt histories had decreased functional connectivity between the amygdala and left ventral and right rostral PFC while viewing emotional faces (Johnston et al., 2017). Other imaging studies of youth with and without histories of suicidal ideation or suicide attempts have also demonstrated altered neural activation in medial and midline regions implicated in processing of negative emotions (Just et al., 2017; Pan et al., 2013). Further, some have found altered neural connectivity within neural networks implicated in executive control among youth with histories of major depressive disorder (MDD) and suicidal ideation, compared to MDD alone (e.g., Ordaz, Goyer, Ho, Singh, & Gotlib, 2018).

Collectively, these preliminary studies suggest that youth with a history of STBs may exhibit altered neural processing of emotional cues. If so, youth experiencing, or at risk for STBs may exhibit exaggerated or blunted responses to negative experiences in everyday life. Importantly, the subcortical brain structures discussed here govern peripheral stress responses discussed below (Berthoud & Neuhuber, 2000; Herman et al., 2003) and may transact with stressors also discussed later in this review. Indeed, chronic underarousal, or poor cognitive control following acute stressors has been suggested as an important pathway for adolescent STBs (Bernanke, Stanley, & Oquendo, 2017; Miller & Prinstein, 2019). Establishing links such as these between systems will be an important direction for research in the next decade.

### Future Directions

Future work in neuroscience-based models of STBs will require attention to several limitations. First, studies to date have been exclusively cross-sectional with small sample sizes. Larger, prospective studies are needed to determine the extent to which neuroimaging studies offer unique value for suicide research. Second, because neuroscience research tools are still developing, findings should be interpreted tentatively until replicated across numerous studies. Future work involving more rigorous research designs is also warranted. Third, many neuroscience methods involve paradigms or experimental tasks that may lack ecological validity or may not accurately simulate experiences in realtime or across real-world settings. Future work that combines multiple methodologies will be important when studying adolescent STB risk (e.g., Oppenheimer et al., 2020).

## PHYSIOLOGICAL RISK FACTORS: HORMONE FLUCTUATION AND SENSITIVITY

Although it is well known that STBs increase dramatically at the adolescent transition, remarkably little research has examined how changes in gonadal hormones may be associated with youth suicide. Preliminary findings in the past decade have suggested that this may be a fruitful line of inquiry, however, perhaps especially for understanding associations between hormone sensitivity and elevated rates of suicide ideation and attempts among adolescent girls. Note that this preliminary work has offered the potential to understand not only which adolescent girls may be at greatest risk for STBs, but also when at-risk girls may be most likely to experience suicide ideation or attempt suicide. Note that some research also has examined associations between adolescent male hormone changes (i.e., testosterone) and STB risk, yielding equivocal results (Perez-Rodriguez et al., 2011; Rice & Sher, 2017; Sher, 2018). In addition, although some males exhibit sensitivity to hormonal fluctuations and corresponding changes in mood similar to patterns in females described below (Schmidt et al., 2004), there has been far more research on females than males to date. Importantly, males do not experience the consistent and predictable acute hormone cycling that is characteristic of the menstrual cycle, and such cycling has shown distinct links to STB. This review below therefore is limited to emerging findings among adolescent girls.

Specifically, using novel methods from the field of reproductive mood disorders, new data in the past decade have begun to clarify how and for whom changing hormone levels may confer increased risk for STBs. Interestingly, gender differences in rates of STBs emerge at the onset of puberty and diminish following menopause (Owens et al., 2020), suggesting that adolescent girls’ increased risk for STBs may be associated with changes in the hypothalamic–pituitary–gonadal (HPG) axis during puberty (e.g., Angold, Costello, Erkanli, & Worthman, 1999, but see Marceau, Ruttle, Shirtcliff, Essex, & Susman, 2015). In fact, emerging research suggests that the weeks both immediately preceding and following onset of menses (i.e., the perimenstrual phase, a period of rapid decline in estrogen and progesterone) may be associated with heightened STB risk for some adolescent girls.

Data supporting associations between the menstrual cycle and STBs have come from two bodies of literature. First, robust evidence suggests that natural menstrual hormone fluctuations modulate known STB risk factors among hormone-sensitive women (for a review, see Owens & Eisenlohr-Moul, 2018). For some adolescents (Buddhabun-yakan et al., 2017; Czajkowska, Drosdzol-Cop, Gałązka, Naworska, & Skrzypulec-Plinta, 2015), the perimenstrual phase of the menstrual cycle is associated with exacerbations in several affective risk factors for STBs, including depressive symptoms, anxiety, and anger/irritability (e.g., Bentley et al., 2016). In addition, as many as 39% of adolescents retrospectively report social impairment in the perimenstrual period (Vichnin, Freeman, Lin, Hillman, & Bui, 2006), and prospective studies of adolescents also suggest increases in angry outbursts and social withdrawal in the perimenstrual period (Buddhabunyakan et al., 2017; Czajkowska et al., 2015). While these studies did not directly examine STBs, disruptions in social experiences have been shown to be strong predictors of adolescent girls’ STBs in other work (e.g., Heilbron & Prinstein, 2010). Second, research recently has examined direct associations between hormone changes across the menstrual cycle and STBs, although such work has primarily been conducted in adults. Cross-sectional studies demonstrate higher rates of suicide attempts and deaths in the weeks prior to and during menses, compared to other menstrual cycle phases (Saunders & Hawton, 2006; Zengin et al., 2015). An important direction for future research will be to examine the extent to which perimenstrual fluctuations in hormones may be associated with acute changes in STBs among adolescents.

Importantly, not all girls are likely to experience clinically significant menstrual cycle-linked fluctuations in mood and behavior. Experimental studies demonstrate that menstrual cycle changes in mood and behavior are driven by abnormal sensitivity to normative hormone fluctuations, rather than unusual levels of ovarian hormones (Schmidt et al., 2017). While only a minority of individuals appear to experience this hormone sensitivity, suicidal adolescents may be at particularly high risk, as several known STB risk factors have also been linked to increased hormone sensitivity. For example, early life adversity and trait-level impulsivity are robustly associated with both history of STBs among adolescents (Auerbach, Stewart, & Johnson, 2017) and menstrual cycle-linked changes in mood and other psychological symptoms (Eisenlohr-Moul et al., 2016). In addition, perimenstrual worsening of symptoms is apparent for several disorders with high rates of STBs, including depressive disorders, borderline personality disorder, and eating disorders (Eisenlohr-Moul et al., 2018; Klump, Keel, Culbert, & Edler, 2008). While large epidemiological studies have yet to establish the exact prevalence of hormone sensitivity among adolescents, small studies suggest that prevalence rates may be comparable to adult populations. For example, one study (*n* = 125) found 8% of adolescents met criteria for premenstrual dysphoric disorder (PMDD), which is characterized by emotional, behavioral, and physical symptoms that cause distress or impairment in the luteal phase (i.e., the two weeks before menses) and dissipate in the follicular phase (Czajkowska et al., 2015). A greater percentage of adolescent girls (42%) demonstrate clinically significant symptoms around menses without meeting full criteria for PMDD (Czajkowska et al., 2015).

### Menarche and Developmental Norms

Studying potential links between the menstrual cycle and STBs is especially relevant to adolescence given the occurrence of puberty during this developmental period. Pubertal timing has been associated with several domains of risk (Ullsperger & Nikolas, 2017), and the age at which adolescents experience menarche (i.e., first onset of menstruation) has important implications for STB risk. Most adolescents experience menarche between ages 12 and 13 (American Academy of Pediatrics Committee on Adolescence, Laufer, & Breech, 2006), and earlier age of menarche is associated with greater engagement in both NSSI and STBs (Deng et al., 2011; Roberts, Fraser, Gunnell, Joinson, & Mars, 2019). Early menarche also is associated with greater hormone sensitivity (e.g., Cohen et al., 2002), which may increase perimenstrual STB risk in girls who experience earlier menarche. Further, girls who experience earlier menarche are more likely to have more ovulatory cycles in the years immediately following menarche (AAP Committee on Adolescence et al., 2006). Given that ovulation is the primary driver of hormone fluctuation across the menstrual cycle (Hammarbäack, Ekholm, & Bäackström, 1991), this greater frequency in ovulatory cycles is likely associated with more frequently symptomatic cycles for hormone-sensitive individuals, which may contribute to increased STB risk for these individuals.

### Future Directions

Exploring fluctuations in STB risk over the menstrual cycle offers potential to advance an understanding of *when* some individuals may be most vulnerable to experience STBs. Although menstrual cycles in adolescence tend to vary in length compared to adulthood, a majority of cycles last between 21 and 45 days and fall within adult norms by the third year following menarche (WHO, 1986). Thus, despite individual-level variability in cycle length, the menstrual cycle may still be a useful predictor of regular, acute changes in STB risk. Yet, several gaps in this literature require further study in adolescent samples. First, few studies of adolescents have used prospective daily symptom ratings to assess symptoms across the menstrual cycle, relying instead on retrospective self-report. Among adults, retrospective reports of menstrual cycle symptoms are inconsistent with prospective reports and may be biased by social expectations of the menstrual cycle (Marván & Cortés-Iniestra, 2001). Prospective studies among adolescents are needed to accurately model changes in symptoms over the menstrual cycle in order to better understand how and when changes in symptoms alter risk for STBs. Second, future studies should incorporate behavioral measures and data from multiple informants whenever possible. Finally, while studying the menstrual cycle in isolation (i.e., independent of other factors) holds promise for advancing understanding of STB risk, the menstrual cycle occurs alongside other STB-relevant physiological systems (e.g., the HPA axis), as discussed below. Studying interactions of these systems may further advance our understanding of why puberty is a period of heightened risk for some individuals, as well as when these individuals are at greatest risk.

## PROXIMAL RISK FACTORS: PHYSIOLOGICAL RESPONSES TO ACUTE STRESS

As noted by Franklin et al., (2017), most research on STB risk is based on cross-sectional associations between trait-level factors and a history of prior STBs. Longitudinal data are rare, and prior work has revealed mostly distal, often nonspecific predictors of STBs. For instance, multiple studies have indicated that psychopathology, especially depression, and lifetime adversity are associated with a history of STBs or may be associated with suicide years or even decades later (Bruffaerts et al., 2010; Nock et al., 2013), yet these distal risk factors are poor markers for identifying who will exhibit suicidal ideation or attempts (Franklin et al., 2017). For example, a minority of youth with depressive symptoms or significant lifetime adversity report suicidal ideation, and even fewer attempt suicide.

An alternate approach for understanding STB risk is to identify proximal risk factors, including maladaptive acute stress responses that occur *in the moments, hours, or days* after a significant life stressor but before STBs develop. Data indicate that suicide attempts among adolescents, and perhaps especially among adolescent girls, frequently follow the experience of an acute interpersonal stressor (Berman & Schwartz, 1990; Hawton, Fagg, & Simkin, 1996). Indeed, experiences of stress and maladaptive stress responses may help to explain the precipitous rise in STBs at the adolescent transition. This may be especially true for interpersonally themed stress. Studies based on interviews or checklist data have revealed that adolescent girls frequently report “interpersonal stressors” (i.e., social isolation, peer victimization, “break-ups”,) as precipitants of suicidal behaviors, or more frequently than nonsuicidal hospitalized controls (e.g., Berman & Schwartz, 1990). In addition, findings have revealed several aspects of stressful interpersonal experiences that are concurrently or longitudinally associated with increases in suicidal ideation or suicide attempts (e.g., peer rejection/victimization, low friendship support) (Massing-Schaffer et al., 2019).

Thus, recent research has begun to examine acute physiological stress responses that may be associated with increased risk for STBs, including physiological markers that may elucidate processes that occur between the moment of acute stress and emergence of STBs. Unfortunately, much of this research has been conducted among adults. For instance, cross-sectional work suggests that adults with a history of suicidal ideation may be more likely to exhibit blunted HPA reactivity compared to healthy controls (Melhem et al., 2016; O’Connor, Green, Ferguson, O’Carroll, & O’Connor, 2017). Similarly, a study of depressed and/or self-harming adolescent girls suggested lower levels of cortisol after a dexamethasone suppression test (i.e., cortisol hyporeactivity) was associated with higher reports of suicidal ideation (Beauchaine, Crowell, & Hsiao, 2015). In our own work with adolescents, findings have revealed that HPA hyperreactivity is associated prospectively with higher risk for suicidal ideation over three months (Giletta, Prinstein, et al., 2015). However, when examined in conjunction with higher-than-usual peer stress measured longitudinally, results show that *hyporeactivity* (i.e., blunted HPA axis reactivity) is a prospective predictor of suicide attempts above and beyond the effects of depression, prior suicidal ideation, and prior suicide attempts (Eisenlohr-Moul et al., 2018).

In addition to the HPA axis, investigators also have examined whether parasympathetic withdrawal (i.e., respiratory sinus arrhythmia (RSA) suppression, indicating a fight-or-flight autonomic nervous system (ANS) stress response) in response to laboratory-induced interpersonal stress may be another marker of acute stress response associated with STBs. Concurrent research suggests that excessive RSA withdrawal is more common among those with, compared to those without, suicide attempt histories (Tsypes, James, et al., 2018; Tsypes et al., 2018. In prospective work, Giletta et al., (2017) revealed that greater RSA withdrawal among adolescent females following a social stressor task was associated with higher levels of suicidal ideation over the following nine months; however, not all findings have been supported longitudinally (Weilgus, Aldrich, Mezulis, & Crowell, 2016).

### Future Directions

Studying physiological responses to acute social stressors offers an opportunity to depart from traditional models examining distal risk factors and instead examine which individuals are most likely to respond to known STB precipitants (e.g., stressful interpersonal experiences) in potentially harmful ways. Thus, this approach offers promise for identifying imminent risk factors and may inform preventive interventions. However, this approach is in its infancy; Franklin et al. report that less than 6% of studies have examined proximal risk factors for STBs, and thus, much more work is needed. Future work might examine a wide range of physiological stress response markers not discussed here (for a review, see Miller & Prinstein, 2019), as well as developmental factors (e.g., gender, race, ethnicity) that may moderate the meaning and salience of specific precipitants of STBs. Research is also needed to better understand how physiological and psychological factors interact in the moments following a social stressor, in order to better understand how behavioral approaches may mitigate the effects of biological risk.

## BEHAVIORAL/PSYCHOLOGICAL FACTORS: NSSI

Over the past 50 years, the predominant focus of suicide research has been the identification of behavioral and psychological factors associated with STBs. As noted above, much of this research has been cross-sectional and conducted in adults, though a growing number of studies have tested factors that temporally precede and may increase risk for STBs (i.e., risk factors), including in adolescent samples. In a recent review of the adolescent STB literature, Cha, Franz, et al., (2018) identified several predictors—including childhood maltreatment, peer victimization, feelings of worthlessness, and low self-worth—prospectively associated with youth STBs, even while controlling for other known predictors (e.g., depression, prior STBs). Still, strong evidence is available for remarkably few risk factors (Franklin et al., 2017), highlighting the need for new approaches to understand STBs as suggested in this review.

Notably, remarkably strong effect sizes have more recently been revealed for NSSI as a risk factor for STBs. NSSI, or purposeful, direct damage to one’s body tissue *without* intent to die (Nock, 2010), is a prevalent behavior among many adolescents. NSSI is a frequently cooccurring but phenomenologically distinct behavior from *suicidal* thoughts or behaviors (Andover, Morris, Wren, & Bruzzese, 2012). In addition, there is some evidence that NSSI may be as strong—or perhaps an even stronger— predictor of STBs than are prior suicide attempts among youth (Asarnow, Baraff, et al., 2011; Asarnow, Porta, et al., 2011; Guan, Fox, & Prinstein, 2012). While research on adolescent NSSI is not new, the past decade has seen a marked increase in research examining NSSI in relation to later STBs (Ribeiro et al., 2016; Franklin et al., 2017; see also Hamza, Stewart, & Willoughby, 2012). Therefore, we focus this section on recent findings and future directions regarding NSSI.

Research in the past decade has attempted to better understand basic descriptive characteristics of NSSI among youth, as well as whether such characteristics predict presence or frequency of STBs. Recent estimates suggest that between 13 and 18% of adolescents and young adults have engaged in NSSI at least once in their life (Muehlenkamp, Claes, Havertape, & Plener, 2012; Swannell, Martin, Page, Hasking, & St. John, 2014), and prevalence rates are significantly greater (i.e., 35–60%) among clinical adolescent samples (Asarnow, Baraff, et al., 2011; Asarnow, Porta, et al., 2011; Wilkinson, Kelvin, Roberts, Dubicka, & Goodyer, 2011). Cutting is one of the most commonly endorsed methods of NSSI, followed by scratching, hitting, and burning, and many individuals use multiple methods (Whitlock et al., 2011). Early research has clearly identified specific psychological functions that may be met by NSSI (for a recent meta-analysis, see Taylor et al., 2018). Certain functions (e.g., affect regulation) have been shown to confer greater risk for continued engagement in NSSI (Pollak, D’Angelo, & Cha, 2020); of note, other functions (e.g., dissociation) show associations with STBs, including suicide attempt histories (Paul, Tsypes, Eidlitz, Ernhout, & Whitlock, 2015).

Importantly, this and other work suggests that particular characteristics of NSSI may predict lesser or greater risk for STBs. For example, more frequent engagement in NSSI is associated cross-sectionally with more frequent suicide attempts (Andover & Gibb, 2010). A recent meta-analysis found that descriptive features of NSSI behaviors, such as frequency of engagement and number of methods, were among the strongest predictors of suicide attempts among those who engage in NSSI (Victor & Klonsky, 2014). Severity and lethality of NSSI injuries (e.g., scarring) also show strong associations with STBs, suggesting that these characteristics may be especially informative indices of STB risk (Burke, Ammerman, & Jacobucci, 2019). Much of this work, however, has been cross-sectional, and further work is needed to better understand key characteristics of NSSI among adolescents. Key characteristics for future examination include frequency across days, months, and years (Paul et al., 2015), type, number, and severity of methods used to engage in NSSI (Victor & Klonsky, 2014), experiences of pain and severity of injuries (Ammerman, Burke, Alloy, & McCloskey, 2016), and associated psychological distress and functional impairment.

Beyond descriptive characteristics and cross-sectional correlates, longitudinal studies in the past decade have begun to more rigorously examine risk factors for NSSI, as well as prospective associations between NSSI and STBs. Regarding risk factors for NSSI, research suggests that a history of physical and sexual abuse, peers’ engagement in NSSI, prior NSSI or STBs, “cluster b” personality types, and hopelessness are among the strongest known predictors of NSSI longitudinally, although effect sizes remain small (see Fox et al., 2015). Further, several studies have now demonstrated that NSSI is associated prospectively with later STBs (Asarnow, Baraff, et al., 2011; Asarnow, Porta, et al., 2011; Guan et al., 2012; Wilkinson et al., 2011). These studies reflect growing empirical interest in examining trajectories and cooccurrence of NSSI and STBs in adolescence (Brunner et al., 2007; Nock, Joiner, Gordon, Lloyd-Richardson, & Prinstein, 2006). For instance, Giletta, Calhoun, et al., (2015), Giletta, Prinstein, et al., (2015) found evidence for joint trajectories of NSSI and suicidal ideation among adolescents over a two-year, multiwave follow-up period, such that youth with chronically high suicidal ideation were at greatest risk of chronically high NSSI, and vice versa. Other recent work has further examined the temporal overlap and direction of associations between NSSI and STBs in adolescents (Glenn, Cha, et al., 2017; Glenn, Lanzillo, et al., 2017; Whitlock et al., 2013). Notably, Glenn et al. (2017) found that transitions between self-injurious thoughts and behaviors (both NSSI and STBs) may occur quickly in adolescence, suggesting that the first 6–12 months after onset of NSSI or suicidal ideation represents a critical window for intervention and prevention of more several suicidal behaviors. Notably, relatively little work has examined how NSSI may be associated with, or may mediate the associations among many of the risk factors discussed above (e.g., neural markers of cognitive control, hormone changes, physiological proximal risk factors) and adolescent STBs.

### Future Directions

More research is needed to better understand why and for whom NSSI is associated with STBs, including under what conditions NSSI may progress to suicidal behaviors. Recent evidence strongly suggests that NSSI is a predictor of later STBs among adolescents and that NSSI often occurs following significant emotional distress; thus, the study of NSSI among researchers interested in emotion regulation would be fruitful. For instance, future research might further examine time-variant changes in affect in the days, minutes, or moments preceding engagement in NSSI. Other priorities in this literature include the need for more large-scale longitudinal research, particularly in community samples, as well as research examining similarities or distinctions between NSSI and STBs (Nock, 2010; Whitlock et al., 2013). This could include studying joint developmental trajectories of NSSI and STBs, as well as clarifying the extent to which these outcomes should be understood as etiologically distinct. Research would also benefit from further examining different “profiles” of NSSI engagement, such as occasional versus chronic engagement. Use of procedures that capture intraindividual fluctuations in predictors (e.g., negative affect) or self-reported motivations for NSSI (e.g., functions) could elucidate more fine-grained, dynamic patterns of NSSI engagement, as well as if and when these behaviors become suicidal in nature (i.e., performed with some intent to die).

## BROADER SOCIETAL FACTORS IMPACTING DIVERSE POPULATIONS

In the last decade, rates of STBs have increased among diverse groups of adolescents. Epidemiological data reveal significant increases in STBs not only among those long known to exhibit high rates of STBs (e.g., White females and Native American youth) but also among groups that have previously been understudied (Lindsey, Sheftall, Xiao, & Joe, 2019), suggesting a need for greater focus on youth that represent racial, ethnic, sexual, and gender diversity. Indeed, as with many areas in developmental research (Galambos & Leadbeater, 2000), prior work on STBs has predominantly examined White, non-Hispanic samples and more rarely minority groups, including sexual and gender minority youth (Cha, Tezanos, et al., 2018). Given emerging research to suggest that predictors of STBs vary considerably within discrete populations, a multilevel (i.e., individual, community, and societal level) framework may be best suited to understand STB risk in diverse populations (National Action Alliance for Suicide Prevention within its Research Prioritization Task Force, 2014; Pena, Matthieu, Zayas, Masyn, & Caine, 2012). Finally, these studies represent growing interest among adolescent STB researchers in intersectionality: variation in risk and resilience factors among diverse minority identities (e.g., sexual orientation, race, gender identity), explored within the context of interlocking systems of oppression and inequality (Cole, 2009; Crenshaw, 1991).

### Theoretical Framework

While some prior adolescent STB research has focused on racial, ethnic, sexual, and gender minority youth, most studies have used methodological frameworks that increasingly feel outdated and inappropriate for examination of diverse populations (e.g., race as “nuisance” variable, dichotomous White vs. non-White analytic approaches). Diverse groups need not be examined in comparison to White, cisgender, or heterosexual peers; examination of within-group variability is sorely needed. Currently, this work is largely limited to specialty journals, increasing the problematic trend of overwhelmingly White samples and maintaining the status quo of White supremacy within psychological research. Research on diverse populations would benefit from identifying culturally sensitive STB risk factors and theoretical models that are specific to the population of interest. Similarly, research on diverse populations should use systems-oriented language (Hong, Espelage, & Kral, 2011) that acknowledges a group’s broader societal context and considers the potential influence of systemic inequities on clinical outcomes that may be present among a particular group.

In this section, we draw on two theories in discussing STB research in diverse populations. First, Meyer’s (2003) minority stress theory suggests that experiences related to stigma, discrimination, and prejudice may exacerbate links between social stressors experienced by minority groups (e.g., lesbian, gay, and bisexual individuals) and adverse physical or mental health outcomes. Adverse social experiences may be especially relevant for understanding STBs (e.g., Durkheim, 1951; Joiner, Van Orden, Witte, & Rudd, 2009), and in the past decade, studies have begun to more directly examine STB models in the context of minority stress theory (Plöderl et al., 2014). Additionally, minority stress theory calls attention to resilience factors that may help explain why rates of STBs among some racial and ethnic minority groups have previously remained lower than among majority peers (Polanco-Roman, Anglin, Miranda, & Jeglic, 2019). Second, research in the past decade suggests that suicide may be best understood within the context of ecological systems theory (Bronfenbrenner, 1992; Hong et al., 2011; Wyatt, Ung, Park, Kwon, & Trinh-Shervin, 2015) that incorporates social determinants specific to STB risk. Environmental context may be particularly pertinent among minority populations (Hong et al., 2011), and adolescent STB researchers have already begun adopting these frameworks (Boyas, Kim, Villareal-Otálora, & Sink, 2019). With these theories in mind, we discuss recent research and new directions for the study of STBs among diverse populations of adolescents.

### Asian American, Native Hawaiian, and Pacific Islander (AA/NHPI) Adolescents

Remarkably little work has examined STBs among AA/NHPI adolescents (Wyatt et al., 2015). The “model minority” myth may partly account for this empirical discrepancy (Chou & Feagin, 2015). Moreover, prior work often examines AA/NHPI youth as a homogenous group, potentially obscuring important variability based on country of origin or regional variation (Islam et al., 2010). However, emerging research in the past decade suggests that lower levels of acculturation and high levels of parent–child conflict are associated with greater risk for STBs among AA/NHPI youth (Lau, Jernewall, Zane, & Myers, 2002). In contrast, research suggests that familial relationships characterized by parental warmth and family cohesion may protect against STBs among this group (Wong & Maffini, 2011; Wyatt et al., 2015). Future work might examine resilience factors unique to AA/NHPI youth, while also considering variability based on country of origin so as not to treat AA/NHPI youth as a monolithically homogenous group.

### Black Youth

The majority of research on Black youth has examined ethnically labeled African American populations. While “Black” is a broader term that may more accurately reflect societal impacts (e.g., systemic racism) in lieu of more specific cultural or ethnic identification, past research has largely used the term “African American”. For the purposes of this review, we focus on African American youth but encourage future research to attend to variability based on racial and ethnic identity among Black youth.

While African American youth traditionally have lower rates of STBs compared to the general population, rates have risen dramatically among this group in the past decade (Kann et al., 2018). Perceived discrimination, racism, and microaggressions may play a role in emergence of STBs among African American adolescents (English, Lambert, & Ialongo, 2014; Lanier, Sommers, Fletcher, Sutton, & Roberts, 2017). These effects may be further compounded for those from lower socioeconomic backgrounds (Chambers & Erasquin, 2018) or who experience weight discrimination or bullying (Garnett et al., 2014). Importantly, some evidence suggests higher levels of school connectedness (Tomek, Burton, Hooper, Bolland, & Bolland, 2018) and emotional self-efficacy may be particularly protective against STBs for African American adolescents (Valois, Zullig, & Hunter, 2015). Given that mental health services are often inaccessible or unavailable in predominantly African American neighborhoods (Barksdale, Azur, & Leaf, 2010), future STB prevention efforts would need to address this disparity, while also addressing mental health stigma that may be heightened in African American communities (Amutah-Onukagha et al., 2018). To this end, researchers have advocated for strengths-based, culturally relevant research that works with community partners to promote resilience, racial empowerment, and access to services in order to reduce STB risk among African American adolescents (Merchant, Kramer, Joe, Venkataraman, & King, 2009; Opara et al., 2020).

### Latinx Youth

Latinx adolescents have lower rates of death by suicide than non-Hispanic Whites, but Latinx individuals are more likely to attempt suicide than their White counterparts (Kann et al., 2018). Indeed, Latinx youth have had the second highest rates of suicide attempts behind Native American youth for the past 40 years (Romero, Bauman, Borgstrom, & Kim, 2018). Like many other groups, individual factors such as isolation, depression, and hopelessness are associated with increased risk for STBs among Latinx youth (Kuhlberg, Peña, & Zayas, 2010; Romero, Edwards, Bauman, & Ritter, 2014); however, the mechanisms by which these factors confer risk may be different. For instance, recent work suggests that familism, connectedness to the local community, and parent–adolescent conflict may be particularly relevant in modulating risk for STBs among Latinx youth (Gulbas, Guz, Hausmann-Stabile, Szlyk, & Zayas, 2019; Silva & Van Orden, 2018; Vega, 2014). Recent preliminary research also suggests that intrinsic religiosity and parental monitoring may have a moderating mediation effect on suicidal ideation through depression for Latinx adolescents (Boyas et al., 2019), whereby both mitigate depressive symptoms and subsequently lessen risk for suicidal ideation. Further study of familial and community-level factors and STB risk in Latinx youth is warranted. Future work might also distinguish between countries of origin to investigate culturally specific differences among Latinx youth.

### Native American Youth

Compared to all other racial and ethnic groups, Native American adolescents are the most likely to attempt and die by suicide (SAMHSA, 2017). Yet, literature on Native American adolescent STBs remains limited (Olson & Wahab, 2006). Disparate rates of STBs for Native American youth, as well as limited engagement in research, likely reflect a long history of marginalization and collective disempowerment among Native American individuals (Kral, Idlout, Minore, Dyck, & Kirmayer, 2011; LaFromboise, Albright, & Harris, 2010). Notably, a majority of Native American youth never receive behavioral health care for active STBs (Freedenthal & Stiffman, 2007). Thus, the study of STBs among Native American adolescents is a high priority for future work, including research focusing on community-level factors or historical contexts that underlie generational mistreatment of Native American populations (Thira, 2014). Recent research on Native American adolescents reveals that suicide attempts may be associated with maltreatment experiences (e.g., sexual violence, victimization) that occur more frequently within Native American communities (Breiding et al., 2014; Wiglesworth & Prinstein, 2021). Further work will likely require community-based participatory research that facilitates trust, collaborative inquiry, shared control, and mutual respect for differences throughout data collection and interpretation (Wexler et al., 2015). Research might also examine culturally specific protective factors that may be relevant in reducing STBs among Native Americans (Harder, Rash, Holyk, Jovel, & Harder, 2012).

### Lesbian, Gay, and Bisexual Youth

Lesbian, Gay, and Bisexual (LGB) youth are two and a half times more likely to attempt suicide than heterosexual youth (King et al., 2008), and adolescence into young adulthood represent periods of highest STB risk for LGB individuals (Marshal et al., 2013). Research suggests sexual minority youth face more victimization, exclusion, and mistreatment at school compared to their heterosexual peers (Burton, Marshal, Chisolm, Sucato, & Friedman, 2013), and experiences of cyberbullying confer even greater risk for STBs among LGB youth (Duong & Bradshaw, 2014). As many as 80% of LGB youth report experiencing verbal harassment, and 40% report physical violence, at school (Kosciw, Greytak, Bartkiewicz, Boesen, & Palmer, 2012)—known risk factors for STBs (Bontempo & D’Augelli, 2002). Studies also point to perceived burdensomeness as a critical mediating factor linking sexual orientation and risk for STBs (Baams, Grossman, & Russell, 2015; Woodward, Gray, Wingate, & Pantalone, 2014). These findings, among a growing body of work on homophobic bullying, suggest that interventions that promote feelings of belonging or support systems may be especially beneficial among this population (Barnett, Molock, Nieves-Lugo, & Zea, 2019; Espelage et al., 2019; Hatchel, Merrin, & Espelage, 2019). Lastly, youth often begin to disclose their sexual orientation to peers and family in adolescence (Martos, Nezhad, & Meyer, 2015). This process may confer additional risk for STBs, especially if “coming out” is received negatively (Haas et al., 2011). On the other hand, coming out may be associated with the presence of protective factors, such as enhanced social support and less internalized homophobia (Plöderl et al., 2014). STB interventions targeting stress related to the coming out process may be more effective than those targeting internalized experiences of homophobia (Michaels, Parent, & Torrey, 2016).

Similar to other minority groups—which are often clustered together for expediency in developmental research—LGB youth vary in their experiences related to STBs (Hatchel, Polanin, & Espelage, 2019). Bisexual youth report poorer mental health outcomes and higher levels of internalized homophobia than their gay and lesbian peers (Kuyper & Fokkema, 2011). Research suggests that “double minority” youth (i.e., adolescents who are sexual minorities with a second accompanying minority identity) experience compounded distress (Hayes, Chun-Kennedy, Edens, & Locke, 2011). In a recent study of American high school students, adolescents with disabilities who also identified as LGB were at highest risk for victimization and suicidal ideation (King, Arango, & Ewell Foster, 2018).

### Transgender and Gender Nonconforming Youth

Transgender youth (i.e., individuals whose gender is different than their sex assigned at birth) are at higher risk for STBs compared to cisgender peers (Meier & Labuski, 2013). Similarly, gender nonconforming youth (i.e., individuals whose gender expression does not match masculine or feminine gender norms) may be at increased risk for STBs (Spivey & Prinstein, 2019). Although often grouped together with sexual minority youth, transgender and gender nonconforming adolescents experience unique vulnerability factors (APA, 2015). Gender identity and gender expression represent distinct components of identity that should not be conflated with sexual identity; therefore, studies that distinguish between gender identity, gender nonconformity, and sexual orientation are needed in STB research (Connolly, Zervos, Barone, Johnson, & Joseph, 2016; Spivey & Prinstein, 2018).

Suicidal thoughts and behavior research among transgender youth should consider multiple risk factors that may result from minority stress. For instance, 60% of transgender students report experiencing verbal harassment or physical violence (Kosciw et al., 2012). This rate is particularly alarming when considering transgender and gender nonconforming youth are four times as likely to attempt suicide following experiences of physical violence, compared to their cisgender peers (Testa et al., 2012). Compared to cisgender peers, transgender and gender nonconforming youth may also experience lower body esteem and higher rates of verbal and physical abuse, both of which may increase risk for future STBs (Grossman & D’Augelli, 2007). On the other hand, transgender and gender nonconforming youth who affiliate with peers who identify similarly to them report significantly less anxiety and STBs, particularly during early stages of identity development (Testa, Jimenez, & Rankin, 2014). Research also highlights the importance of friend support during identity development in reducing STB risk for transgender youth (Tebbe & Moradi, 2016), and further studies of peer- and community-related resilience factors are needed (Espelage et al., 2019).

### Future Directions

While adolescent STB research over the past decade has made strides in incorporating minority stress and ecological systems theories, more longitudinal research is needed to examine developmental trajectories of STBs among diverse groups of youth. As mentioned above, consideration of inter-sectionality is critical. Among Latinx youth, for example, high variability in rates of STBs based on sex, sexual orientation, immigration status, region of residence, and other identifiers suggests that consideration of diversity cannot be limited to any singular marker of minority status (Klonsky, May, & Saffer, 2016; Romero et al., 2018). Critically, multiethnic sexual minority youth, when compared to other sexual minority subgroups, may be most likely to experience STB risk factors (Craig & McInroy, 2013; O’Donnell, Meyer, & Schwartz, 2011). Yet, intersectionality-focused research among diverse adolescent populations remains relatively rare (Abrutyn & Mueller, 2019; Torres, Mata-Greve, Bird, & Hernandez, 2018).

Future adolescent STB work should continue to examine subgroup differences that account for diverse experiences of minority stress, understood within broader community- and systems-level contexts (O’Brien, Putney, Hebert, Falk, & Aguinaldo, 2016). This work might move beyond studies of group differences toward designs that consider the influence of structural factors (e.g., resource allocation, power hierarchies) that may adversely affect minority groups (Grzanka & Miles, 2016). Such approaches include community-based participatory research that engages group strengths and encourages collaboration with the communities being studied. Moreover, interdisciplinary “team science” approaches may be necessary to accrue the sample sizes needed to study within-group differences and community-informed risk models. Finally, prevention and treatment of STBs within minority populations will also require culturally tailored approaches that engage both individual- and systems-level factors.

## ADDITIONAL FUTURE DIRECTION

Several additional areas of research deserve attention to help stimulate increased study of adolescent STBs. Each is discussed briefly below. In some cases, this research has advanced considerably in the past decade, suggesting a fruitful avenue for ongoing work.

### Real-Time Monitoring

Real-time monitoring techniques, also known as ecological momentary assessment (EMA) or experience sampling, offer a critical methodological advancement for understanding temporal dynamics, immediate precipitants, contextual correlates, and within-person fluctuations in STBs. These methodologies permit collection of many *intra*individual data points across time and settings (e.g., beyond the lab) but are relatively new in the STB literature. Recently, real-time monitoring approaches have become more feasible. In fact, youth may be uniquely amenable to this mode of data collection (Wen, Schneider, Stone, & Spruijt-Metz, 2017), and research suggests these approaches are safe and feasible for studies of adolescent STBs (Kleiman, Glenn, & Liu, 2019).

EMA holds promise for addressing important gaps in the STB literature, such as clarifying the timescale across which STBs unfold within individuals. Emerging evidence suggests that suicidal ideation shows as much or greater *intra*individual (i.e., across hours of the day) compared to between-person variability (Kleiman et al., 2017), perhaps especially among adolescents who report shorter episodes of ideation (Nock et al., 2009). Moreover, EMA can help differentiate low-level suicidal ideation from episodic peaks (i.e., suicidal crises) and identify affective states associated with time-lagged changes in STBs (e.g., sadness; Husky et al., 2014). Recent studies harnessing technology-assisted data collection (e.g., use of wearables) have also revealed associations of STBs with fluctuations in biological regulatory processes such as circadian rhythms (e.g., sleep schedules; Littlewood et al., 2019). Future research should continue to attend to ethical and safety practices when conducting ecological monitoring studies among those experiencing, or at risk for, STBs (Nock, Kleiman, Abraham, Bentley, & Brent, 2020).

### Complex Risk Models and Machine Learning

Research that incorporates complex models of risk may meaningfully advance prediction of adolescent STBs, especially in light of evidence that even the most potent predictors of STBs show relatively weak predictive accuracy (i.e., only slightly greater than chance; Ribeiro et al., 2016). Recent research testing complex machine learning algorithms has yielded promising results. A recent review of machine learning techniques and STBs concluded that studies applying machine learning to predict STBs show greater predictive accuracy compared to studies using traditional statistical methods (Burke et al., 2019). Among adolescents, several predictive algorithms (e.g., logistic regression, lasso, ridge, random forest) have been shown to predict suicide attempts. Depending on ease of implementation and availability of data inputs, these algorithms may be practical and clinically useful (Miché et al., 2020). Further, work by Hill, Oosterhoff, and Kaplow (2017) and Walsh, Ribeiro, and Franklin (2018) suggest that data from routine clinical examinations may prospectively predict adolescent STBs and could be incorporated into screening efforts to reduce nonfatal suicide attempts. Machine learning approaches hold promise for improving the accuracy, scalability, and clinical utility of tools to predict adolescent STBs, and more research and replication of prior results are needed.

Practical and ethical barriers to implementation will also need to be addressed. Resistance to machine learning, in the form of clinician skepticism or time and cost barriers (e.g., need for provider training), may hinder its implementation in clinical settings (Kilsdonk, Peute, & Jaspers, 2017). Further, while models demonstrate preliminary success in predicting *who* is at risk for STBs, future advancements must also address the question of *when* to intervene (Linthicum, Schafer, & Ribeiro, 2019). More work is also needed to clarify how best to tailor interventions or clinical approaches based on information from algorithmic models. Lastly, machine learning algorithms remain imperfect modeling techniques, thereby raising ethical considerations. False positives (i.e., inaccurately labeling an individual as “at risk”) could result in misallocation of resources; more consequentially, false negatives (i.e., inaccurately labeling an individual as “not at risk”) may have fatal consequences. Modeling errors raise further questions about liability and whether the algorithm, model developers, or healthcare personnel should be held responsible for the consequences of such errors remains ethnically ambiguous (Linthicum et al., 2019). Future researchers must attend to these ethical questions as they continue to advance the complexity of risk models and consider their implementation in applied settings.

### Social Media

Given widespread social media use among adolescents, research has begun to more rigorously examine the effects of social media on depression, anxiety, and, increasingly, STBs among youth. Much of this work has focused on cybervictimization, cyberbullying, and “suicide contagion”. Being a victim of cyberbullying is associated with suicidal behaviors (for a review, see John et al., 2018), and research on “suicide contagion” suggests that adolescents often learn of peers’ suicide attempts on social media. Further, exposure to suicide stories online may be associated with elevated suicidal ideation (Dunlop, More, & Romer, 2011; Robertson, Skegg, Poore, Williams, & Taylor, 2012). Other recent work has explored the potential benefits of social media use for adolescents experiencing, or at high risk for, STBs. Online social engagement may protect against STBs by promoting self-disclosure and help-seeking behaviors (Frost & Casey, 2016; Lewis & Seko, 2016), while online friendships have been shown to offer protective benefits against future suicidal ideation among adolescents (Massing-Schaffer, Nesi, Telzer, Lindquist, & Prinstein, 2020). Some work suggests that online communication platforms are more often constructive than detrimental for youth who engage in NSSI or STBs (Daine et al., 2013).

Despite these advances, evidence of causal links between social media use and STBs in adolescence remains mixed (Ophir, Lipshits-Baziler, & Rosenberg, 2019; Twenge, Joiner, Rogers, & Martin, 2018). Most studies of social media use and adolescent mental health outcomes—including, but not limited to, STBs—have yielded small associations that do not necessarily support causality (Odgers & Jensen, 2020). Research on adolescent internet usage, and STB outcomes specifically, shows similar patterns, with roughly equal numbers of studies finding positive, negative, and mixed associations (Dyson et al., 2016; Marchant et al., 2017). More research is needed exploring the ways social media may either buffer against or exacerbate STBs among youth. Future work might examine STBs specifically in the context of unique features of social media (e.g., availability of social information, opportunities for anti-normative behaviors; see Nesi, Choukas-Bradley, & Prinstein, 2018) that have transformed the frequency, immediacy, and publicness of adolescents’ social interactions.

### Sleep

In the past decade, researchers have increasingly focused on sleep characteristics (i.e., sleep problems, such as insomnia, hypersomnia, abnormally short or long total sleep durations, nightmares, and circadian reversal) as potential risk factors for STBs. Sleep holds promise as an intriguing focus in suicide research for several reasons. First, sleep problems have long been considered warning signs for STBs given associations with heightened arousal or dysregulation, states often linked to proximal STB risk in theoretical and empirical work. Second, because sleep can be measured objectively (e.g., using mobile wearable devices) and may show intraindividual fluctuation over short time periods, it offers promise as a proximal STB risk factor. Third, sleep (i.e., quality and quantity) is malleable and can, with effective intervention, be regulated and improved.

Reflecting interest in this area, a handful of reviews of sleep and STBs have been published in the past 10 years. Findings suggest STBs are associated with at least some types of sleep problems (e.g., Bernert, Kim, Iwata, & Perlis, 2015; Pigeon, Pinquart, & Conner, 2012). However, most work has been conducted among adults using cross-sectional designs. A recent meta-analysis of adult- and adolescent-based longitudinal studies found evidence for prospective associations between sleep problems and STBs, with small-to-medium effect sizes (Liu et al., 2020). However, results should be interpreted within important caveats; for example, a majority of longitudinal studies featured long follow-up intervals, leaving uncertain whether sleep problems predict short-term STB risk.

A smaller number of studies have examined longitudinal associations between sleep problems and STBs specifically among adolescents (for a review, see Kearns et al., 2020). For example, Wong, Brower, and Zucker (2011) found that sleep problems in early adolescence predicted suicidal ideation three years later, even when controlling for prior suicidal ideation and depressive symptoms. Sleep problems such as insomnia and nightmares also have been shown to predict changes in suicidal ideation over shorter intervals of 7–21 days (Bernert, Hom, Iwata, & Joiner, 2017). Similarly, insomnia and hypersomnia have been shown to predict suicide attempts over multiyear follow-up, although not all findings have held when controlling for other risk factors such as hopelessness (Wong & Brower, 2012); Nrugham, Larsson, & Sund, 2008). Overall findings are mixed but suggest a positive association between sleep problems and STBs in youth. Future research should examine specific (e.g., initial insomnia), rather than more general (e.g., poor sleep), sleep problems prospectively in relation to STBs, ideally over short follow-up intervals to establish their short-term predictive validity. Future work is encouraged to integrate objective measures of sleep quality or quantity (e.g., sleep variability, measured via actigraphy) and pursue longitudinal designs in both clinical and community samples of youth (Kearns et al., 2020).

### Genetics

Few studies have examined the potential genetic underpinnings of STBs among adolescents. While no reviews to our knowledge have focused on the genetics of STBs specifically in youth, reviews of case–control, family-based association, and genome-wide association studies across both adult and adolescent samples point to several genes that may be linked to suicidal behaviors, although results are mixed and inconsistent across studies (e.g., see Mirkovic et al., 2016 for a review). Research testing main effects of particular candidate genes, for example, has revealed modest support for associations between suicidal behaviors and several serotonergic and neurotrophic factor genes (Li & He, 2006; Zai et al., 2012). Other work has examined whether certain genes might moderate associations between environmental factors (e.g., childhood adversity) and STBs (i.e., gene–environment interactions; see Duncan, Pollastri, & Smoller, 2014). This research posits that particular genotypes may confer risk in the presence of environmental risks, perhaps especially during key developmental periods such as adolescence. One longitudinal cohort study found evidence for an interaction of serotonergic genes (i.e., HTR2A variants) with histories of sexual and physical abuse in predicting suicide attempts, including in adolescents (Brezo et al., 2010). However, a majority of genetic work on STBs has been conducted in adult samples, with largely inconclusive results that have yet to be replicated (Duncan et al., 2014; Mirkovic et al., 2016). It also remains difficult to disentangle risk conferred by genetics or heritable factors from that conferred by shared environmental factors associated with STBs.

Currently, there is an emphasis on genome-wide research regarding genetic risk (National Institute of Mental Health, 2017); past work on candidate genes and adolescent suicide may be useful to guide hypothesis generation in future work. Among adolescents, early research provided preliminary evidence for the heritability of STBs through adoption, twin, and family-based genetic association studies (for reviews, see Brent & Mann, 2005; Brent & Melhem, 2008). Much of this work examined rates of STBs among relatives of adolescents who had attempted or completed suicide. For example, Brent’, Bridge, Johnson, and Connolly (1996) widely cited study of adolescent suicide probands showed an increased rate of suicide attempts among first-degree relatives of adolescents who died by suicide compared to controls, even when controlling for psychiatric disorders. Similarly, a prospective investigation in a mixed-age sample of adolescents and adults found a fivefold increased odds of suicide attempt among offspring of parents with suicide attempt histories (Brent et al., 2015). More recently, a handful of studies have taken gene-candidate or genome-wide association approaches to identify or test specific genes or genetic variants that may modulate risk for STBs in adolescents. Brent et al., (2010) found evidence linking the FKBP5 gene (i.e., a genotype associated with glucocorticoid receptor subsensitivity) with new-onset or worsened suicidal ideation or behaviors in depressed adolescents. These findings suggest a potential role of genes involved in processes that may decrease HPA axis sensitivity to feedback or alter cortisol secretion, which is consistent with tentative evidence for associations between genes linked to inflammatory responses and suicidal behaviors in older or mixed-age samples (Clive et al., 2016; Galfalvy et al., 2015; Guintivano et al., 2014). Further, Zalsman et al. found associations between MAOA genotypes and STBs among suicidal adolescents presenting at, or admitted to, the hospital (Zalsman et al., 2011), but did not find significant effects for 5-HTR2A (i.e., a serotonergic candidate gene) in another investigation (Zalsman et al., 2005). A recent study found that specific haplotypes within HTR2C and ANKK1-DRD2 genes, which are implicated in serotonergic and dopaminergic pathways, significantly altered risk for suicidal ideation in adolescence depending on individuals’ genetic background (Hill, Jones, & Haas, 2020). Despite these findings, no specific genes or genetic risk factors have conclusively been shown to predict STBs in adolescents. Prior findings require replication, and more research is needed to more precisely identify genetic markers associated with STBs in adolescence.

### Resilience

While research to date has not identified many robust risk factors that increase risk for STBs (Franklin et al., 2017), the study of resilience factors, perhaps especially among researchers with expertise in adolescence, may prove fruitful for identifying factors that buffer the effects of STB risk factors. Research examining resilience factors has gained considerable traction in the adult STB literature (e.g., see Kleiman & Anestis, 2015) but requires replication in adolescent samples. Additionally, while a majority of this work has focused on intrapersonal protective factors (e.g., grit, gratitude, self-esteem; Johnson, Wood, Gooding, Taylor, & Tarrier, 2011), more recent work in youth has begun to consider a broader range of systems- or environment-level protective factors, such as family unit, peer relationships, school context, and community environment (Gallagher & Miller, 2018). For example, a recent study of adolescent females found evidence for protective effects of high levels of parental and peer support against STBs following interpersonal distress (Mackin, Perlman, Davila, Kotov, & Klein, 2017). These effects did not hold for noninterpersonal stress, suggesting that resilience factors may differentially influence STB risk depending on the type of stressor, as well as ecological context and other factors unique to diverse groups (Gallagher & Miller, 2018). Future work should therefore consider the interaction of individual, cultural, and ecological resilience factors among diverse populations to further clarify the range of factors that may protect adolescents from STB onset.

### Empirically Supported Treatment

Research has identified surprisingly few well-supported, effective treatments for adolescent STBs. While existing treatments for related psychopathology, such as cognitive-behavioral therapy (CBT) for depression, have shown marginal reductions in STBs (i.e., via coping techniques and affect regulation skills; Spirito, Esposito-Smythers, Wolff, & Uhl, 2011), novel treatments for adolescent STBs are limited. Moreover, a recent meta-analysis of interventions for STBs found overall small treatment effects and poor efficacy across randomized controlled trials, with even weaker intervention effects among child and adolescent populations (Fox et al., 2020). Nevertheless, several important advances in treatment research from the past decade can inform future work in this critical area. First, it is known that the time immediately following discharge from psychiatric hospitalization represents a particularly high-risk period for STBs among adolescents (Chung et al., 2017). Given that adolescents may not receive sufficient care following discharge (Spirito, Simon, et al., 2011), research efforts have specifically targeted this critical posthospitalization period as a point of intervention. For example, family-based support coaching interventions have been shown to reduce postdischarge STBs, when compared to treatment-as-usual approaches (Asarnow, Baraff, et al., 2011; Asarnow, Porta, et al., 2011).

Second, growing bodies of work advocate for use of psychosocial interventions that target general and specific outcomes on the spectrum of self-injurious thoughts and behaviors that proceed suicide. For instance, dialectical behavior therapy for adolescents (DBT-A; Miller, Rathus, & Linehan, 2006) appears to meet criteria as a well-established treatment for the reduction of self-harm and suicidal behavioral in adolescents, compared to individual and group supportive therapy (IGST) and treatment-as-usual approaches (McCauley et al., 2018). DBT-A may be particularly effective among adolescents with high levels of emotion dysregulation and parental psychopathology (Adrian et al., 2019). Additionally, preliminary studies utilizing DBT Coach mobile applications (Rizvi, Hughes, & Thomas, 2016) have been shown to reduce subjective experiences of distress among suicidal adolescents. These and other mobile-based interventions warrant further study, especially given their potential to circumvent several limitations of full-scale DBT-A and other traditional modes of treatment delivery (e.g., see Kazdin, 2019). For suicidal ideation, attachment focused family-based therapy (FBT-A) and interpersonal therapy (IPT) show efficacy in reduction of suicidal ideation, while family-focused CBT with parent training demonstrates the most evidence for suicide attempts (Glenn, Franklin, & Nock, 2015). Importantly, as recently emphasized in greater detail in meta-analyses by other research groups (Fox et al., 2020; Glenn et al., 2015), this remains a pressing need.

Further promising advancements in STB intervention work over the past decade include the development and testing of integrative treatment designs that engage relevant support figures and target comorbid diagnoses. Asarnow and Mehlum (2019) concluded that the most efficacious interventions address adolescents’ psychosocial environment and train relevant adult figures in supportive care. Indeed, interventions such as Safe Alternatives for Teens and Youths (SAFETY; Asarnow, Berk, Hughes, & Anderson, 2015)—which is rooted in social-ecological models of behavior change and incorporates elements of CBT, DBT, and family therapy—may have considerable promise in reducing STBs among high-risk adolescents (Asarnow, Hughes, Babeva, & Sugar, 2017). Similarly, the Youth-Nominated Support Team Intervention for Suicidal Adolescents (YST) employs an adolescent-nominated “support team” of adults who receive ongoing psychoeducation in how to best support high-risk adolescents (King et al., 2019). More specialized treatments, such as integrated outpatient CBT interventions for cooccurring alcohol and/or drug use and suicidality (i.e., I-CBT), have demonstrated efficacy in reduction of both substance use and STBs (Esposito-Smythers, Spirito, Kahler, Hunt, & Monti, 2011). Such findings further support a need for interventions that address the interplay of multiple harmful and reinforcing behaviors (King, Merrin, Espelage, Grant, & Bub, 2018). For all aforementioned treatments, work requires further validation and application to diverse populations; further research and treatment innovation is needed to address, develop and test interventions for adolescent STBs (see recent work from Fox et al., 2020; Glenn et al., 2015 for further review).

## CONCLUSION

This review provides an extensive, though still limited summary of advances in adolescent STB research over the past decade. We outline laudable efforts to identify, test, and further explore STB risk factors across neuroscientific, biological/physiological, behavioral/psychological, and broader societal domains. We also touch upon technological advances that offer promise for enhanced measurement and prediction of STBs (e.g., real-time monitoring, machine learning) and create new digital environments for communication and social interaction that may alter risk for STBs among adolescents (e.g., social media). Finally, while we focus primarily on research examining factors and processes associated with onset or maintenance of STBs, other recent work has focused on how best to address STBs once they emerge (e.g., empirically supported treatments).

In light of these findings, future research should consider multiple developmental systems to better understand why the adolescent period, or the pubertal transition in particular, is associated with such dramatic increases in rates of STBs. Notably, there has been remarkably little research examining transactions between developmental systems that confer risk for STBs, despite the likelihood that main effect models are insufficient for understanding these complex behaviors. Further, we encourage future research to consider the role of cultural, racial/ethnic, and systems-level factors in STB risk, as well as whether and how STBs vary within diverse populations. Finally, where possible in this review, discrete STB outcomes were discussed to illustrate the spectrum of thoughts and behaviors that precipitate suicide. Such distinctions are critical, particularly with the growing knowledge of “ideation-to-action” theories of suicide (for recent work, see Klonsky, Saffer, & Bryan, 2018) that suggest differing risks, resilience factors, and interventions based upon the unique STB in question. Future research would be prudent to distinguish between types of STB outcomes to both further enhance our knowledge of how these thoughts and behaviors function in adolescence, as well as continue to improve prevention and intervention efforts during this sensitive period of development.

In the coming decade, adolescent STBs will remain a critical research focus for psychological scientists. While safety concerns may have previously deterred some researchers from pursuing STB-related questions, there now exist several protocols to guide safe STB research among both community and clinical samples of youth (e.g., Helms & Prinstein, 2014). STBs in adolescents are complex phenomena tied to developmental trajectories and implicating multiple psychological domains (e.g., affective, social, cognitive). Further advances will require collaboration among interdisciplinary teams to better understand emergence and maintenance of these perplexing, often fatal outcomes in adolescence.

## Figures and Tables

**FIGURE 1:**
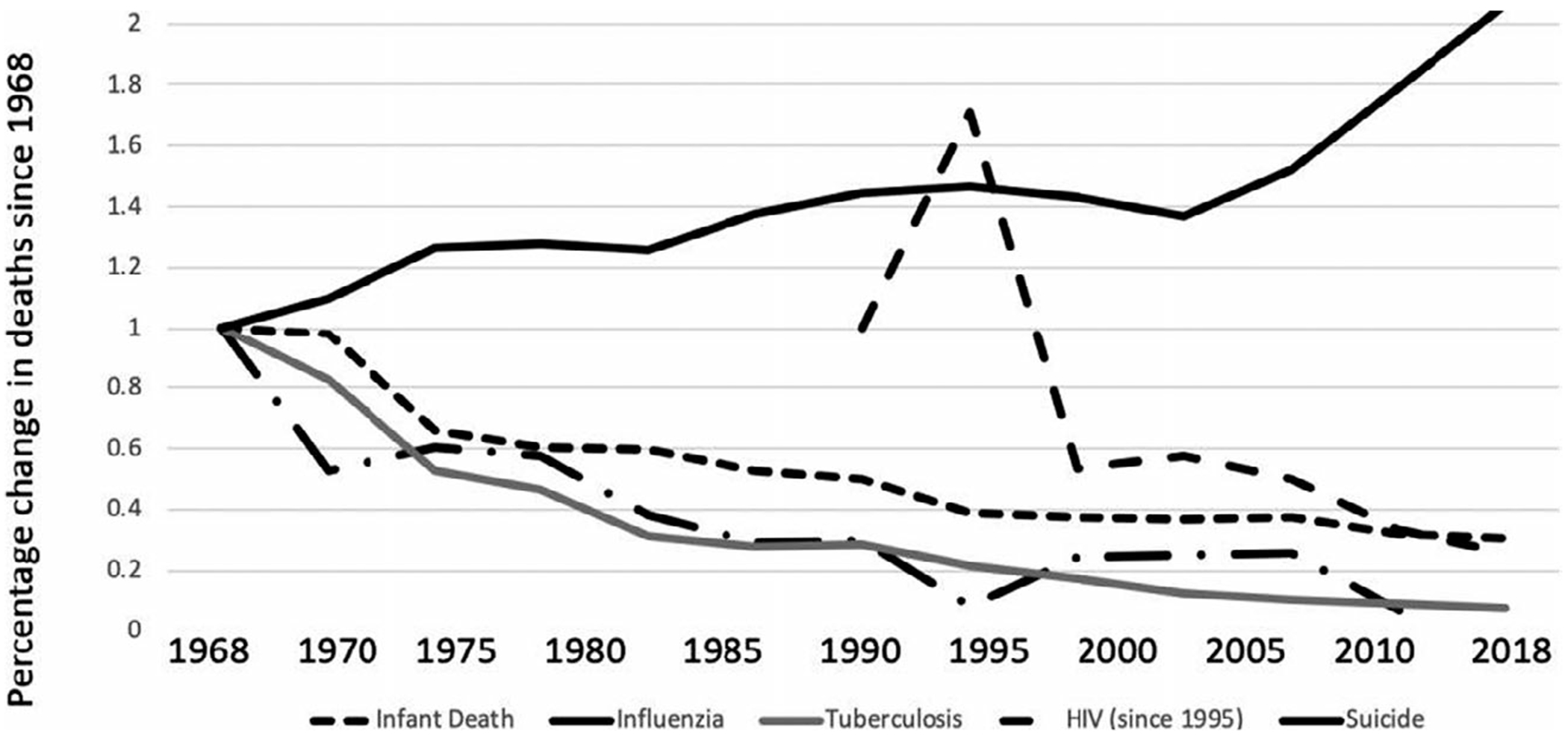
Leading causes of death over the past 50 years. *Note:* Data represents the leading causes of death over the past 50 years as a proportion of change in death rate since 1968. Data adapted from the Centers of Disease Control and Prevention WONDER Online Database (2019).

**FIGURE 2:**
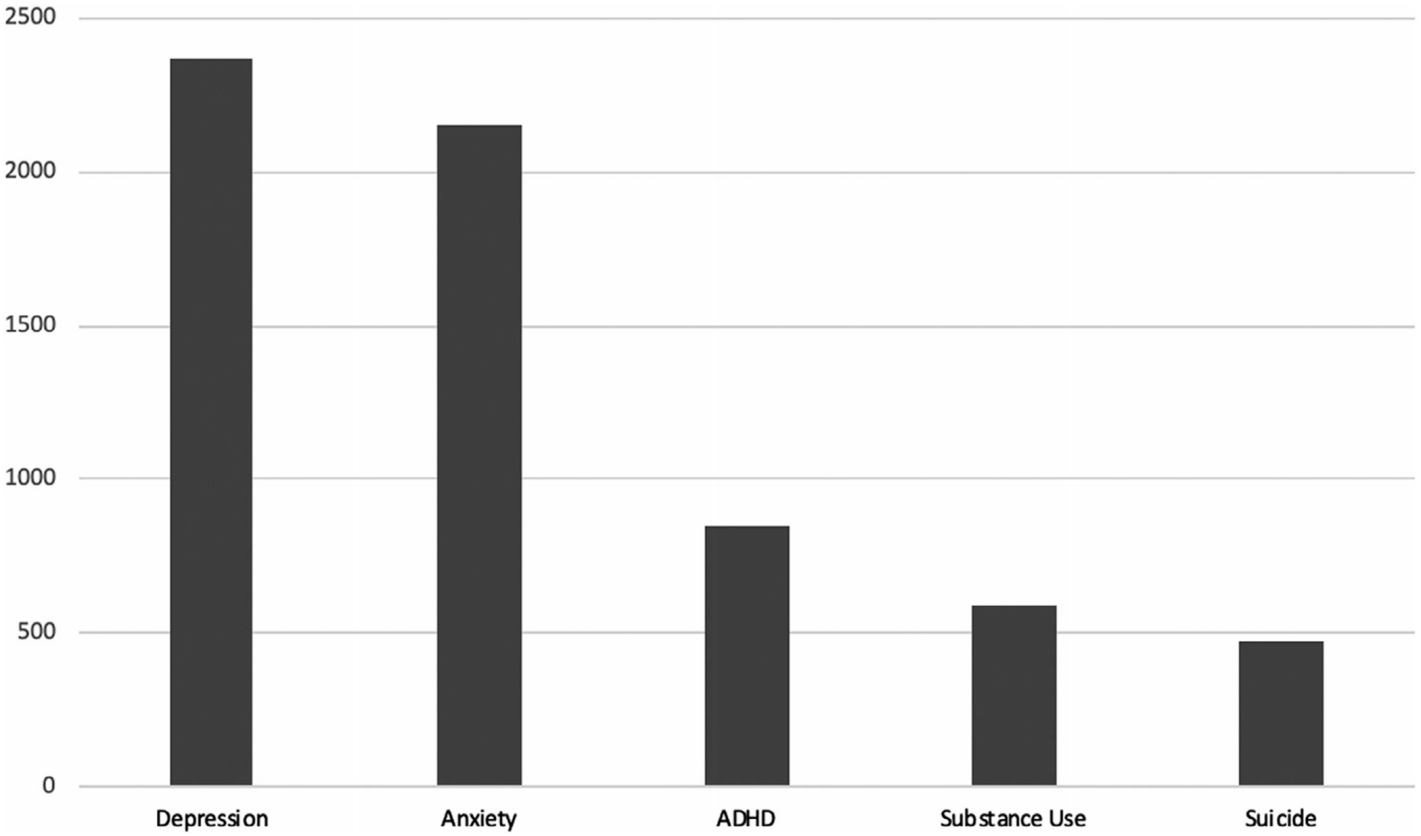
Publications in high impact journals for several adolescent clinical problems from 2011 to 2020. *Note:* Data represents results of PsychInfo keyword search utilizing expanders and Boolean/Phrase search modes across 20 high impact developmental and clinical psychology journals (all impact factors > 4.000), limited to the adolescent age period (13–17). Search completed using the following terms for respective clinical problems: depression (“depress*),” anxiety (“anxiety”), ADHD (“adhd or attention of deficit hyperactivity disorder or attention deficit-hyperactivity disorder”), substance use (“substance abuse or substance use or drug abuse or drug addiction or drug use”), and suicide (“suicide*”). This table did not preclude overlapping cases.
